# Acute lung injury induced by acute uremia and renal ischemic-reperfusion injury: The role of toll-like receptors 2 and 4, and oxidative stress

**DOI:** 10.22038/IJBMS.2022.64025.14099

**Published:** 2022-05

**Authors:** Seyedeh-Sara Hashemi, Sahar Janfeshan, Zeinab Karimi

**Affiliations:** 1 Burn and Wound Healing Research Center, Shiraz University of Medical Sciences, Shiraz, Iran; 2 Shiraz Nephro-Urology Research Center, Shiraz University of Medical Sciences, Shiraz, Iran

**Keywords:** Acute lung injury, Acute kidney injury, Bone marrow, Inflammation, Mesenchymal stem cell, Oxidative stress, Pattern recognition - receptors

## Abstract

**Objective(s):**

Acute lung injury (ALI) is a common complication of distant organ dysfunction induced by acute kidney injury (AKI). Toll-like receptors (TLRs) have a critical role in progression of AKI. The main goal of this study was to determine whether lung gene expression of TLR2 and TLR4 change by ischemic (renal bilateral ischemic-reperfusion; BIR) and uremic (bilateral nephrectomy; BNX) AKI.

**Materials and Methods:**

Forty male rats were divided into five groups. Two kidneys were removed in BNX, and renal pedicles were clamped in BIR for 45 min. The kidney and lung tissue, and blood samples were collected and saved after 24 hr in all groups. The bone marrow mesenchymal stem cells were immediately injected (1×10^6^,IV) into the treated groups. The expression of TLR2, TLR4, TNF-α, and VEGF was checked by RT-PCR in the tissue samples. MDA level, SOD, and CAT activity were evaluated in the tissue samples.

**Results:**

Structural disturbance of ALI was detected as alveolar hemorrhage and vascular congestion after BIR and BNX. Lung TLR2 and TLR4 but not TNF-α and VEGF up-regulated in these groups. Oxidative stress stabilized after the BIR and BNX in the tissue samples. BMSCs reduce the expression of TLR2 and TLR4 and oxidative stress in the treated groups.

**Conclusion:**

Acutely gathering systemic mediators after renal ischemic or uremic injury induce ALI through overexpression of TLR2 and TLR4 and oxidative stress. Therefore, the Lung protective effect of BMSCs may be related to modulation of TLR2 and TLR4 and oxidative stress in the kidney and lung tissue.

## Introduction

Despite advances in dialysis and other unique treatments for critically ill patients, survival is reduced in AKI, and the mortality rates are higher than 50% (1). AKI has a high frequency of systemic consequences, mainly lung injury (2), contributing to increased mortality. The mechanism of respiratory complications in patients with AKI is not completely understood. By animal models, we and other researchers have previously indicated that lung injury occurs following ischemic AKI or nephrectomy (3, 4). Bilateral nephrectomy is used to separate the special effects of renal failure (impaired clearance and metabolism) from those of renal ischemic-reperfusion (I/R). Some studies showed that AKI in rodents induced pulmonary damages, characterized by alveolar edema and hemorrhage, leukocyte infiltration, and capillary leak (5, 6). 

TLRs are a family of germline-encoded pathogen-recognition receptors that have been evolutionarily conserved from the worm *Caenorhabditis elegans* to mammals (7). Many studies have demonstrated a conserved family of TLRs as critical devices for tissue injury (8, 9). Some TLRs are expressed on several immune cells, such as dendritic cells, macrophages, NK cells, B and specific kinds of T cells, and even on the surface of nonimmune cells such as renal tubular epithelial cells mesangial and fibroblasts cells in response to injury (10-12). The expression of TLRs is not static, but it is a dynamic action that is modified quickly in response to pathogens and environmental stresses. Damaged cells release molecules (damage-associated molecular patterns (DAMPs) that stimulate TLRs and cause intercellular activation of transcription factors regulating the expression of many genes or pro-inflammatory cytokines and chemokines (13). 

Our previous study and others showed that expression levels of TLR2 and TLR4 increased after renal ischemic-reperfusion. In addition, in a model of lung ischemia-reperfusion, capillary permeability, the activity of myeloperoxidase, and leukocyte content in bronchoalveolar lavage (BAL) fluid reduced in TLR4-deficient mice (14). Moreover, the severity of lung injury decreased in the absence of TLR4–dependent signaling, including suppression of either phosphorylation and activation (JNK, NF-κB, and AP-1) or expression of pro-inflammatory cytokines (KC, TNF-α, and MIP) in the BAL fluid (15).

During renal ischemia, hypoxia causes tubular epithelial cell damage by increasing the production of reactive oxygen species (ROS), which is excessive in the reperfusion phase of ischemic reperfusion injury (16). On the other hand, the lung is a vulnerable site for oxidative stress. This organ receives a high concentration of oxygen, which along with its large respiratory area and total cardiac output make it susceptible to damage induced by ROS (15). Production of free radicals increases the epithelial cells’ injury by lipid peroxidation of the membrane lipids and oxidative stress damage of DNA and proteins due to cell death (17). Also, the activity of antioxidant enzymes has an essential role in the severity of the injury in ischemia and reperfusion time because they are the first-line of defense against oxidative stress (18). Therefore, oxidative stress is a dangerous condition that advances tissue damage and consequently results in many illness states.

Mesenchymal Stem Cells (MSCs) are a population of self-renewable cells widely available in many tissues, including the bone marrow, adipose tissue, cord blood, brain, liver, muscle, dental pulp, and skin (19, 20). Mesenchymal stem cells isolated from the bone marrow (BMSCs) and adipose tissue are the primary sources for clinical treatment, such as kidney damage (21). These cells do not express blood group antigens (MHC-II or co-stimulatory factors), so they are suitable for allogeneic applications. Previous studies have shown the efficacy and safety of mesenchymal stem cells in treating acute kidney injury (22). The therapeutic role of mesenchymal stem cells is performed through paracrine secretion of the growth factors, cytokines, mitogens, anti-apoptotic, anti-inflammatory, and angiogenic and vasculogenic factors (23). 

Therefore, this study aimed to assay gene expression of TLR2 and TLR4 in the lung tissue in two experimental models of AKI. Also, it examined the lung-protective effect of mesenchymal stem cells through modulation of TLRs and oxidative stress.

## Materials and Methods

***Experimental animals***

The experimental protocols provided by the Ethics Committee of Shiraz University of Medical Sciences were employed to do all experiments. This study was performed on 40 male Sprague-Dawley rats weighing between 260 and 280 gr. The rats were purchased from the animal center of Shiraz University of Medical Sciences. Animals were housed in single cages at a controlled temperature of 22 to 25 °C with lighting (12 hr light/dark cycles) and had access to water and food without restriction. 

***Induction of acute kidney injury***

In this study, the animals were divided randomly into five groups (n=8): sham, BIR (bilateral ischemic-reperfusion), BNX (bilateral nephrectomy), BIR+BMSCs (bone marrow mesenchymal stromal cells), and BNX+BMSCs groups. They were anesthetized with Ketamine (50 mg/kg; Woerden, Netherlands) and xylazine (10 mg/kg; Alfazyne, Woerden, Netherlands) during all interventions. Bilateral renal ischemia and nephrectomy have been described in previous studies (3). Briefly, a midline abdominal incision was done, and the kidney was exposed. Two kidneys were removed in the BNX group, but in the BIR group, the bilateral renal artery and vein were clamped by a microaneurysm vascular clamp for 45 min. After the termination of surgery, the clamps were removed and verified for adequate restoration of the blood flow to the ischemic kidney. The abdominal wound was then closed by 4/0 stitches in 2 layers. In the sham group, the surgical procedure was performed without pedicel clamping. Rats in the BIR+BMSCs and BNX+BMSCs groups received BMSCs (IV, 1×10^6^) immediately after termination of renal ischemia or nephrectomy.

***Experimental protocol***

All animals were re-anesthetized 24 hr after reperfusion. A blood sample was taken, and immediately centrifuged; the plasma was preserved at -20 °C for the quantity of blood urea nitrogen (BUN) and creatinine (Cr). Subsequently, the kidney was harvested; following thoracotomy, it was done rapidly to remove lung lobes. The left kidney and left lobe of the lung were stored at -80 °C for oxidative and molecular assay. The right kidney and right lobe of the lung were maintained in 10% formalin for hematoxylin-eosin staining.

***Isolation and culture of BMSCs***

BMSCs were isolated from rats. Animals’ femurs were cut on both ends and were flushed with Hanks’ balanced salt solution (Invitrogen). Bone marrow cells were cultured in culture flasks under standard conditions using Dulbecco’s Modified Eagle’s Medium (DMEM, Invitrogen) with FBS (Gibco, USA), L-glutamine (Invitrogen), and penicillin/streptomycin (Invitrogen). MSCs were evaluated by flow cytometry (BD Pharmingen, BD FACSCalibur, United States) on the 3rd passage to assess the expression of the mesenchymal surface markers for CD105 and CD90, and hematopoietic surface markers of CD34 (Dako, Denmark), and also between the 3^rd^ and 4^th ^passages were used for the mentioned experiments.

***Kidney function evaluation***

Creatinine and urea nitrogen concentrations of the plasma samples were measured in the laboratory of Nemazi Hospital, Shiraz, using an autoanalyzer (Model RA-1000Technicon, USA).

***Oxidative assay in the kidney and lung tissues***

Oxidative stress was assayed in the homogenized renal and lung tissues using a microplate reader (America, Biotek EPOCH2TC). The activity of catalase (CAT) was measured spectrophotometrically by using the Aebi method (24). Superoxide dismutase (SOD) activity was assayed by Zell Bio Gmbh (Germany (CAT.NO: ZB-SOD 48A). Malondialdehyde (MDA), as the terminal product of lipid peroxidation, was evaluated by the TBARS assay method (25).

***Molecular assay in the kidney and lung tissues***

Total RNA extraction by Trizol (YTzol, Tehran, Iran), measuring purity and concentration of RNA by using NanoDrop™ (Thermo Scientific™, USA) at 260/280 nm, and cDNA synthesis on the basis of manufacturer’s instructions (Add Script cDNA synthesis kit, AddBio Inc., Korea) were performed for each rat′s kidney and lung sample. 

 TNF-α, VEGF, TLR2, and TLR4 transcript levels in rats with and without BMSCs injection and in the sham group were determined, using an in-house SYBR green real-time PCR protocol by Step One Real-Time Instrument (ABI, Step One Plus, Foster City, CA, USA). Here the GAPDH gene was considered an internal control. The reverse and forward primer sequences for transcript amplification were: GDPDH F:5´ AGTGCCAGCCTCGTCTCATA 3´, R: 5´ GAGAAGGCAGCCCTGGTAAC 3´ TNF-α: F:5′ AACA

CACGAGACGCTGAAGT3′, R:5′ TCCAGTGAGTTCC 

GAAAGCC3′, VEGF: F:5′GTCACCGTCGACAGAACA

GT3′, R:5′GACCCAAAGTGCTCCTCGAA3′, TLR2: F: 5´

TGTTCCGGGCAAATGGATCA3´, R:5´GCCTGAAGTG

GGAGAAGTCC 3´ and TLR4: F: 5´TATCCAGAGCCGTT 

GGTGTTAT 3´, R: 5´AATGAAGATGATGCCAGAGCG 3. The real-time PCR mix was composed of PCR master mix (Add SYBR Master, AddBio Inc., Korea), forward and reverse primers (10 pmol), and template cDNA. The real-time program was mentioned in Table 1. Melting curves of the target and internal control genes were analyzed to confirm the specificity of PCR reactions. All real-time tests were repeated twice.

***Kidney and lung histological evaluation***

Kidney and lung tissue samples were fixed in 10% formalin solution. Sections of the tissues were prepared in 5 µm thickness and stained with the H&E method. Renal histopathological injury scoring was performed separately using light microscopy in ten fields (400 magnification) in the cortex and medullary area. The renal damages were characterized by widening of the Bowman’s space, shrinking of glomerular tuft, tubular necrosis, exfoliation of the epithelial cells, cast deposition, and vascular congestion. The lung structural disturbance was evaluated based on the grade of vascular congestion, alveolar hemorrhage, and interstitial thickening. The level of each manifestation was scored according to the changes involved, scoring 0 without changes, 1 with less than 20%, 2 with 20–40%, 3 with 40–60%, 4 with 60–80%, and 5 with greater than 80% (26). The sum of all numerical scores in each group was taken as the total histopathological score. The scoring of lung and kidney histological sections was performed in a blinded and unbiased fashion.

***Statistical analysis***

Statistical analysis was performed between groups using the one-way ANOVA with the Tukey *post hoc* test. The lung and kidney histopathological scores were examined by the non-parametric Kruskal–Wallis multiple comparison test, and a non-parametric Mann-Whitney test was used to show statistical differences between groups. All data were analyzed with GraphPad Prism software 9 to compare the results of the five groups. The *P*-value<0.05 was considered statistically significant. 

## Results

***Characterization of BMSCs***

**Figure 1:**
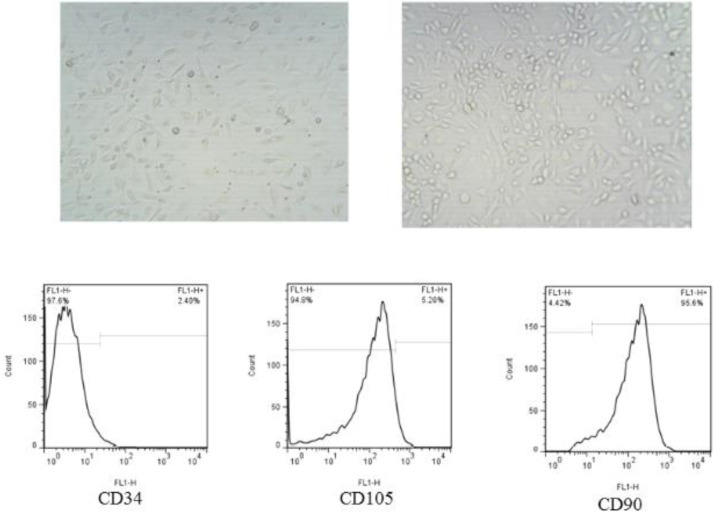
Cytomorphology and flow cytometry of BM-MSCs. BMSCs from passage 3

Morphologically, bone marrow formed a monolayer with fibroblast-like morphology to the culture flasks (Figure 1). The flow cytometry results demonstrated that the cells were expressed positive for mesenchymal markers including CD105 and CD90, and negative for hematopoietic markers of CD34 (Figure 1).

***Renal functional variables after AKI***

**Figure 2:**
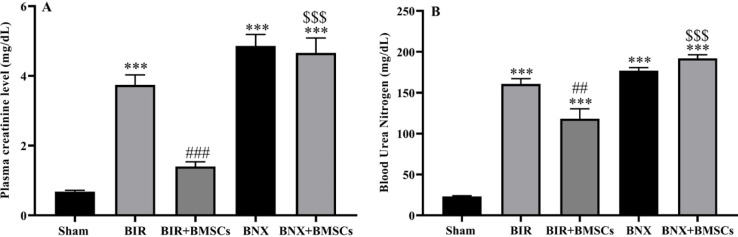
Plasma creatinine (A) and blood urea nitrogen (B) levels after acute kidney injury. These renal functional markers were evaluated 24 hr after reperfusion in sham, non-treatment (BIR and BNX), and treatment (BIR+BMSCs and BNX+BMSCs) groups; n=8 male rats in each group. One-way ANOVA was done with the Tukey *post hoc* test. Data are expressed as mean ± SEM. ***P<0.001 represents significant difference vs the sham group. ##*P*<0.01 and ###*P*<0.001 represent significant difference between BIR and BIR+BMSCs or BNX and BNX+BMSCs groups. $$$ *P*<0.001 represents significant difference between BIR and BNX or BIR+BMSCs and BNX+BMSCs groups

BIR: Bilateral ischemic-reperfusion; BNX: Bilateral nephrectomy; BMSCs: Bone marrow mesenchymal stem cells

Renal dysfunction was induced by bilateral nephrectomy and renal ischemic-reperfusion after 24 hr. This effect was followed by elevation in Cr and BUN compared with the sham group (in both groups, *P*<0.001). However, administration of BMSCs decreased the serum levels of these parameters in the BIR+BMSCs group, but not in BNX+ BMSCs (Figures 2A and B).

***Renal oxidative status after AKI***

**Figure 3:**
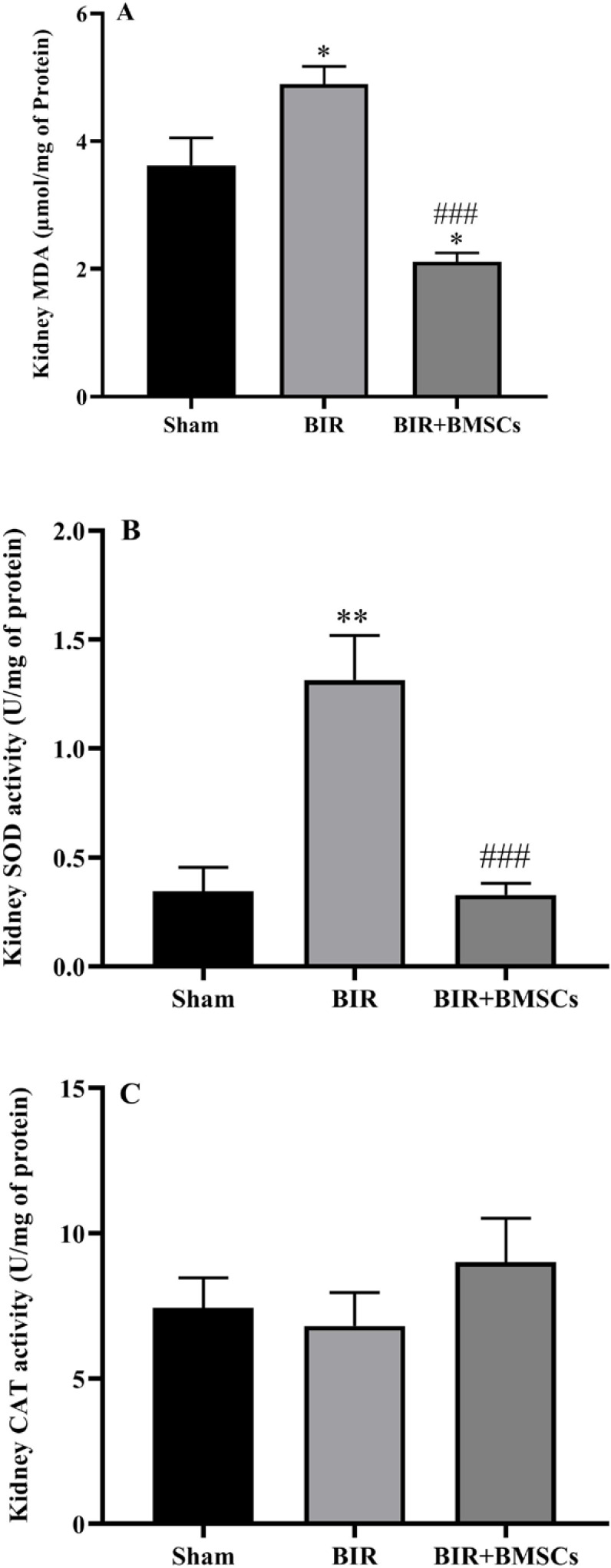
Renal oxidative status after acute kidney injury. The kidney MDA level (A), SOD (B), and CAT activity (C) were measured 24 hr after reperfusion in sham, non-treatment (BIR), and treatment (BIR+BMSCs) groups; n=8 male rats in each group. One-way ANOVA was done with the Tukey *post hoc* test. Data are expressed as mean ± SEM. *P<0.05 and **P<0.01 represents significant difference vs the sham group. ###*P*<0.001 represents significant difference between BIR and BIR+BMSCs

MDA: Malondialdehyde; SOD: Superoxide dismutase; CAT: Catalase; BIR: Bilateral ischemic-reperfusion; BMSCs: Bone marrow mesenchymal stem cells

Renal ischemia following perfusion caused oxidative stress, and this condition exacerbated the acute kidney injury. Our data showed that kidney MDA content significantly increased in the BIR group as compared with the sham group (Figure 3A). Compensatory to an oxidative stress condition, the activity of SOD significantly increased (*P*<0.01), but CAT activity did not change significantly in the BIR group compared with the sham group. Administration of BMSCs modulates oxidative stress. MDA level statistically decreased in the BIR+BMSCs group compared with the BIR group (*P*<0.001). Moreover, CAT and SOD activity respectively increased and reduced after treatment with BMSCs (Figures 3A-C).

***Pulmonary oxidative status after AKI***

**Figure 4:**
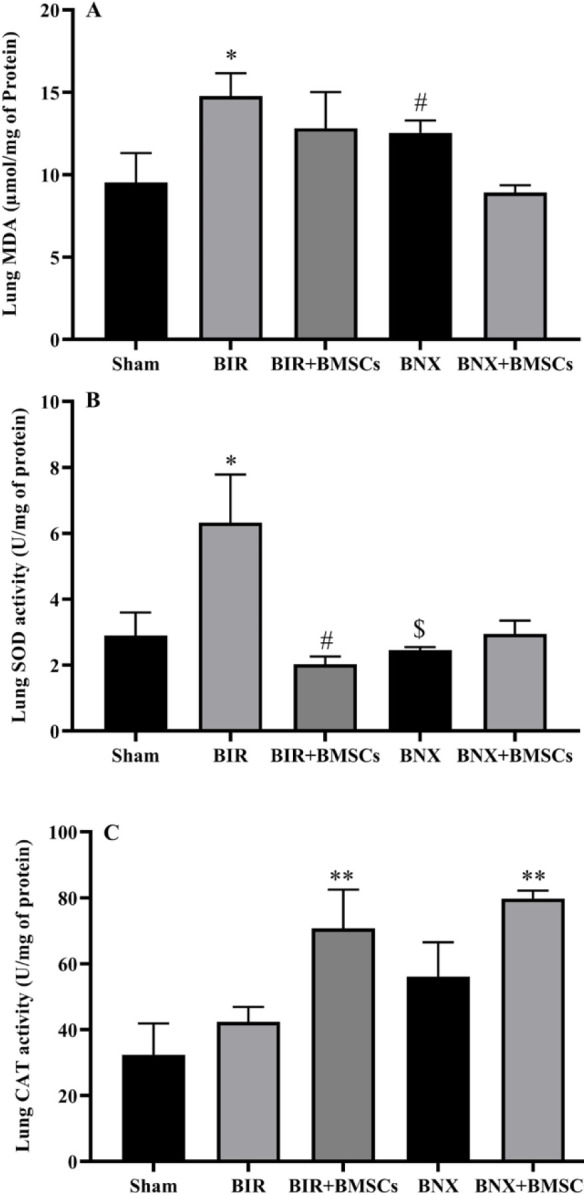
Lung oxidative status after acute lung injury induced by acute kidney injury. The lung MDA level (A), SOD (B), and CAT activity (C) were measured 24 hr after reperfusion in sham, non-treatment (BIR and BNX), and treatment (BIR+BMSCs and BNX+BMSCs) groups; n=8 male rats in each group. One-way ANOVA was done with the Tukey* post hoc* test. Data are expressed as mean ± SEM. *P<0.05 and **P<0.01 represent significant difference vs the sham group. #*P*<0.05 represents significant difference between BIR and BIR+BMSCs or BNX and BNX+BMSCs groups. $*P*<0.05 represents significant difference between BIR and BNX or BIR+BMSCs and BNX+BMSCs groups

MDA: Malondialdehyde; SOD: Superoxide dismutase; CAT: Catalase; BIR: Bilateral ischemic-reperfusion; BMSCs: Bone marrow mesenchymal stem cells; BNX: Bilateral nephrectomy

We investigated pulmonary oxidative status in two models of AKI. The content of MDA was higher in BIR and BNX groups than in the sham group. The activity of SOD and CAT increased in response to oxidative stress in BIR compared with the sham group. While the increase in CAT was not significant. In the BNX group, activity of SOD did not change relative to the sham group but significantly decreased compared with the BIR group. In this group, CAT activity partially increased compared with the sham group. Administration of BMSCs partially decreased the MDA level and increased CAT activity in two treatment groups (BIR+MBSCs and BNX+MBSCs) compared with non-treatment groups (BIR and BNX). While CAT activity significantly increased in two treatment groups compared with the sham group. In comparison, SOD activity did not change relative to the base value after administering BMSCs in both treatment groups. So, activity of SOD significantly decreased in BIR+MBSCs compared with the BIR group (Figures 4A-C). 

***mRNA expression of TLR4, TLR2, VEGF, and TNF-α in the kidney tissue after AKI***

**Figure 5:**
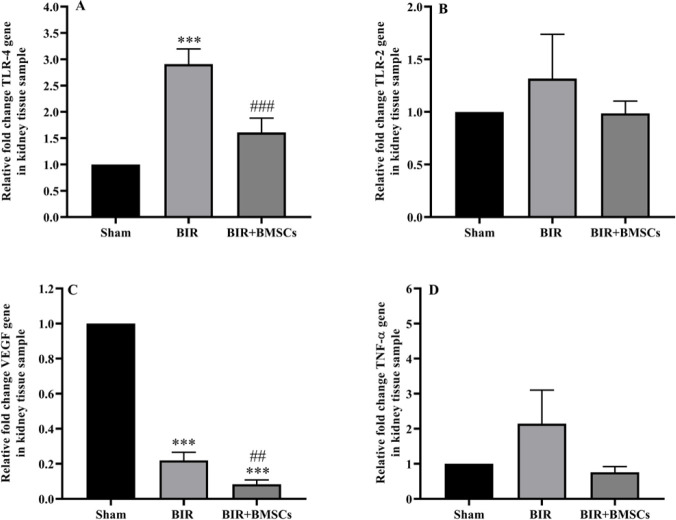
mRNA levels of TLR4 (A), TLR2 (B), TNF-α (C), and VEGF (D) in the kidney tissue after acute kidney injury. Gene expression of TLR2, TLR4, TNF-α, and VEGF was assessed by real-time PCR 24 hr after reperfusion in sham, non-treatment (BIR), and treatment (BIR+BMSCs) groups; n=8 male rats in each group. One-way ANOVA was done with the Tukey *post hoc* test. Data are expressed as mean ± SEM. ***P<0.001 represents a significant difference vs the sham group. ##*P*<0.01 represents a significant difference between BIR and BIR+BMSCs

TLRs: Toll-like receptors; VEGF: Vascular endothelial growth factor; BIR: Bilateral ischemic-reperfusion; BMSCs: Bone marrow mesenchymal stem cells

Gene expression of TLR4, TLR2, VEGF, and TNF-α was measured by real-time PCR. In the BIR group, the mRNA level of TLR4 was significantly higher than in the sham group. In addition, mRNA levels of TLR2 and TNF-α partially increased in the BIR group compared with the sham group. However, the mRNA level of VEGF was significantly lower in the BIR group compared with the sham group. BMSCs treatment down-regulated gene expression of TLR4, TLR2, VEGF, and TNF-α in the BIR+BMSCs group (Figures 5A-D). 

***mRNA expression of TLR4, TLR2, VEGF, and TNF-α in the lung tissue after AKI***

**Figure 6:**
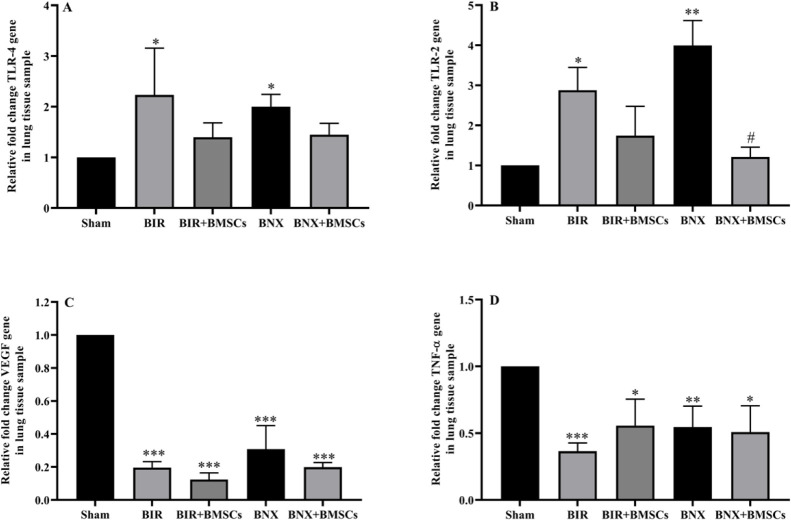
mRNA levels of TLR4 (A), TLR2 (B), TNF-α (C), and VEGF (D) in the lung tissue after acute kidney injury. Gene expression of TLR2, TLR4, TNF-α, and VEGF was assessed by real-time PCR 24 hr after reperfusion in sham, non-treatment (BIR and BNX), and treatment (BIR+BMSCs and BNX+BMSCs) groups; n=8 male rats in each group. One-way ANOVA was done with the Tukey *post hoc* test. Data are expressed as mean ± SEM. *P<0.05, **P<0.01, and ***P<0.001 represent a significant difference vs the sham group. #*P*<0.05 represents a significant difference between BIR and BIR+BMSCs or BNX and BNX+BMSCs

TLRs: Toll-like receptors; VEGF: Vascular endothelial growth factor; BIR: Bilateral ischemic-reperfusion; BNX: Bilateral nephrectomy; BMSCs: Bone marrow mesenchymal stem cells

Following acute kidney injury, pulmonary TLR4 and TLR2 up-regulated significantly in non-treatment (BIR and BNX) groups compared with the sham group but decreased in the treatment (BIR+BMSCs and BNX+BMSCs) groups in comparison. Nevertheless, the mRNA levels of VEGF and TNF-α decreased significantly in treatment and non-treatment groups compared with the sham group (Figures 6A-D). 

***Kidney histology after AKI***

**Figure 7:**
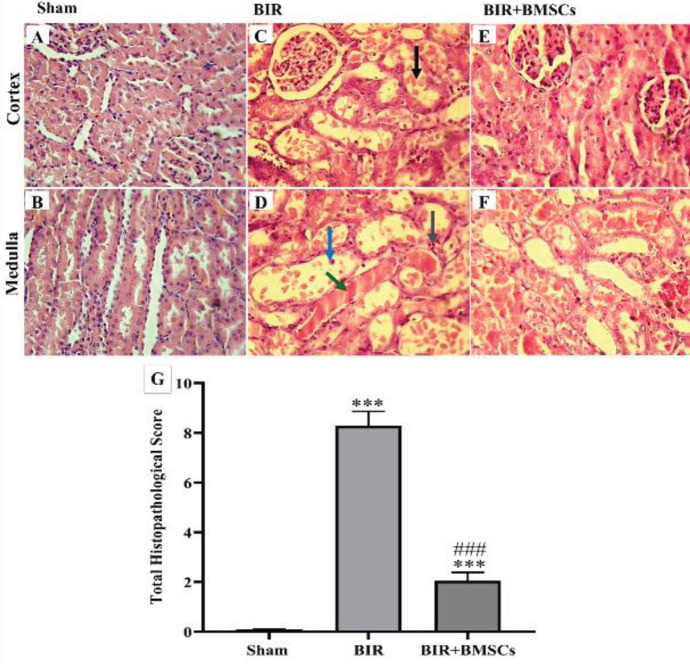
Histological examination of the kidney by H&E staining. 45 min renal ischemia by 24 hr reperfusion caused kidney damage in the cortex and medulla area in the BIR group (C and D) as compared with the sham group (A and B). Administration of BMSCs (IV, 10^6^ cells) decreased the shedding of the brush border (black arrow), acute tubular necrosis (blue arrow), vascular congestion (gray arrow), cast deposition (green arrow), and total histopathological score in BIR+BMSCs group (E, F, and G); n=8 in each group. Nonparametric Kruskal-Wallis was done with the Mann-Whitney test. Data are expressed as mean ± SEM. ***P<0.001 represents significant difference vs the sham group. ###*P*<0.001 represents significant difference between BIR and BIR+BMSCs or BNX and BNX+BMSCs

BIR: Bilateral ischemic-reperfusion; BNX: Bilateral nephrectomy; BMSCs: Bone marrow mesenchymal stem cells

The tubular and vascular appearance was normal in the sham group (Figures 7 A and B), but the renal ischemia following reperfusion caused tissue damage including acute tubular necrosis, shedding of the brush borders, and widening of the bowman space, tubular cast, and vascular congestion. The medullar area is sensitive to hypoxia. Subsequently, the severity of these injuries is higher in the medulla area compared with the cortex area in the BIR group (Figures 7C and D). Moreover, the total histopathological score of the BIR group was significantly (*P*<0.001) higher compared with the sham group (Figure 7G). While administration of BMSCs immediately after ischemia improved the tubular and vascular damages and decreased the total histopathological score of injury in the BIR+BMSCs group (Figures 7E and F, and D, respectively). 

***Lung histology after AKI***

**Figure 8:**
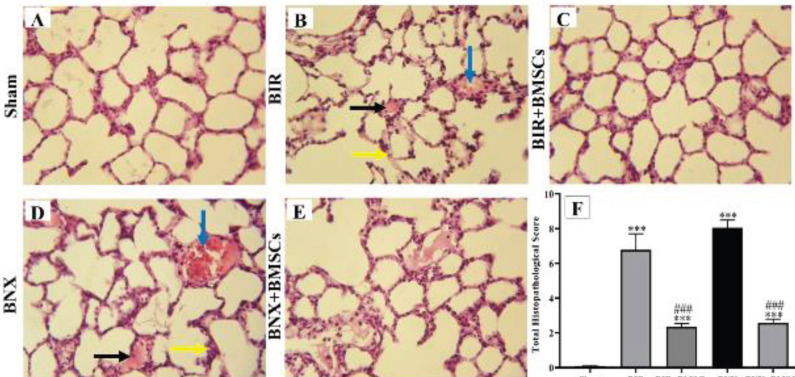
Histological examination of the lung tissue after acute kidney injury by H&E staining (magnification*400). 45 min renal ischemia by 24 hr reperfusion induced lung damages in BIR and BNX groups (B and D) as compared with the sham group (A). Administration of BMSCs (IV, 10^6^ cells) decreased alveolar edema (black arrow), vascular congestion (blue arrow), interstitial thickness (yellow arrow), and total histopathological score in BIR+BMSCs and BNX+BMSCs groups (C, E, and F); Nonparametric Kruskal-Wallis was done with Mann-Whitney test. Data are expressed as mean ± SEM. ***P<0.001 represents a significant difference vs the sham group. ###*P*<0.001 represents a significant difference between BIR and BIR+BMSCs or BNX and BNX+BMSCs

BIR: Bilateral ischemic-reperfusion; BNX: Bilateral nephrectomy; BMSCs: Bone marrow mesenchymal stem cells

Pulmonary histopathology was used to determine the distant organ effect of AKI. The alveolar structure was normal in the sham group (Figure 8A). While lung damages were visibly detected in BIR and BNX groups (Figures 8B and D) as alveolar hemorrhage (accumulation of red blood cells inside the alveoli), increased interstitial thickness, and vascular congestion. Administration of BMSCs (IV, 10^6^ cells) improved lung appearance and total histopathological score in BIR+BMSCs and BNX+BMSCs groups (Figures 8C, E, and F). 

**Table 1:** Real-time program for GAPDH, TNF-α, VEGF, TLR2, and TLR4 transcript amplification

Cycles	Time	Temperature (°C)	Stages
1	30 sec	95	Holding
40	5 sec	95	Denaturation
	20 sec	58	Annealing
	30 sec	72	Extension
1	15 sec	95	Melting
	1 min	58	
	15 sec	95	

VEGF: VEGF: Vascular endothelial growth factor; TLRs: Toll-like receptors

displayed a spindle-shaped morphology. The results of cell surface markers showed that more than 98% of cells were positive for mesenchymal markers of CD105 and CD90, while less than 2% of them were negative for hematopoietic markers of CD34

BMSCs: Bone marrow mesenchymal stem cells

## Discussion

In the present study, 45 min of bilateral renal ischemia with 24 hr reperfusion induced pathological damages such as shedding of the brush border, exfoliation of the cells, acute tubular necrosis, bowman capsule dilation, and vascular congestion in the cortex and medullary area. Renal I/R induced tubular and vascular damage in the kidney (27, 28). Disruption of the cytoskeleton of cells, intracellular binding proteins, and integrin of the tubular epithelial cells lead to loss of cell polarity and shedding of the brush borders (29, 30). Exfoliation of the epithelial cells, shedding of the brush borders, dead cells, and proteins, especially Tamm-Horsfall, produce casts in the lumen of the nephron. These casts obstruct the lumen, restricting the fluid flow from the tubules, increasing the pressure inside the Bowman’s capsule, and dilating the urinary space (31, 32). The most intense tubular damage occurs in the proximal tubule and the thick ascending segment in the outer medullary region, which is hypoxemic even under normal conditions (33).

Renal function was evaluated by measuring the functional biomarkers (creatinine and blood urea nitrogen) after BIR and BNX. Similar to Hoke *et al*.’s study (4), our study showed that plasma levels of creatinine and urea increased at 24 hr after bilateral renal ischemia and bilateral nephrectomy.

During ischemia and following reperfusion, damage-related molecules are released by injured cells that act as endogenous danger signals to stimulate the expression of Toll‐like receptors (34). Recent studies demonstrated that overexpression of TLR2 and TLR4 has a crucial role in facilitating kidney damage after renal I/R (8, 35). In agreement with this study, our data have shown that expression levels of TLR2 and TLR4 increased in the kidney after 24 hr reperfusion. Activation of TLR2 and TLR4 by dangerous ligands promotes the signaling pathways of pro-inflammatory cytokines such as TNF-α (36). The current study also indicated that the mRNA level of TNF-α significantly overexpressed after renal I/R. Vascular endothelial growth factor (VEGF) is a proangiogenic factor, whose expression is triggered by hypoxia in a HIF1-dependent manner (37). Our results show that the mRNA level of VEGF was reduced 24 hr after reperfusion in the BIR group. David *et al*. observed that expression of VEGF was significantly decreased at 1–3 days during renal I/R. However, gene expression of heme oxygenase-1 (HO-1), a gene suggested to be modulated by HIF1 was increased over a period of 1–7 days in response to I/R (38). 

After renal I/R, ROS production is significantly increased, which is an essential pathogenic mediator in progressing cellular damage. MDA is measured as a final product of lipid peroxidation and indicates an index for assessing oxidative stress. Our results were similar to the data by Ma *et al*. (39) who showed that MDA levels increased after renal I/R. In our data, the activity of SOD increased, but CAT activity decreased in the BIR group. The superoxide dismutase enzyme is the first defense line for scavenging endogenous free radicals (40). In this study, the activity of SOD may be compensatory increase against oxidative stress during renal ischemic-reperfusion. However, the CAT enzyme is partly used to remove the excessive free radicals, and hence the activity of this antioxidant is partially decreased in the BIR group. Ma *et al*. showed that activity of SOD and CAT enzymes decreased after renal ischemic-reperfusion (39).

***Acute lung injury induced by AKI***

There is cross-talk between the lung and kidney in both health and illness (41). In confirmation with our previous study (3), the current study showed that lung structural injury is characterized by alveolar edema, increased interstitial thickness, and vascular congestion after two models of acute kidney injury, BIR and BNX. 

Acute lung injury induced by AKI is a multifactorial disease whose mechanism remains incompletely understood (2). Here we wanted to recognize a probable mechanism of acute kidney injury–induced acute lung injury by kidney injury (BIR) or kidney removal (BNX) in the animal models. 

Renal ischemic-reperfusion promotes both renal and extrarenal organ damage through the overproduction of various pro-inflammatory cytokines, oxidative stress, and complement mediators. On the other hand, in the BNX technique, we removed two kidneys, and clearance of systemic mediators such as uremic toxins completely stopped. Therefore, Bilateral nephrectomy is a model to investigate directly the harmful systemic effects of absent renal clearance in AKI without the confusing effects that are related with renal ischemia-reperfusion injury (42).

 Gene expression fluctuation of TLR2 and TLR4 in the lung tissue was assayed. it was demonstrated that bilateral renal ischemia and bilateral nephrectomy could increase the mRNA expression of TLR2 and TLR4 in the lung tissue. 

Several studies found that the serum levels of the many inflammatory mediators (IL-6, IL-1β, IL-12, TNF-α, and G-CSF) increased after ischemic AKI and bilateral nephrectomy, which produced either the renal tubular damaged cells after renal I/RI or extrarenal cells (T lymphocytes) after nephrectomy (4, 16, 43). In our study, local inflammation was confirmed with elevation in the gene expression of TNF-α in the kidney after bilateral renal ischemia-reperfusion. Wolf *et al*. showed that mRNA expression of TLR2 and TLR4 were wholly dependent on the activity of the IFN-γ and TNF-α (44). Therefore, there is a positive cycle between the action of TLRs and pre inflammatory mediators. Although the lung expression of TNF-α was down-regulated in BIR and BNX groups, systemic inflammation and reduced renal clearance of inflammatory factors, creatinine and urea, may contribute to overexpression of TLR2 and TLR4 in the lung tissue after BIR and BNX. In addition, expression of VEGF was down-regulated in this study. Many studies have proposed that VEGF may contribute to the progress of non-cardiogenic alveolar edema (45).

The lung is exposed to the atmospheric air through a massive surface area. On the other hand, the pulmonary tissue has the largest capillary plexuses in the body, which obtain the total right ventricular cardiac output. Furthermore, the lung is susceptible to high levels of O_2_, which make it vulnerable to damage induced by ROS. Oxidative stress is affected by an oxidant/anti-oxidant imbalance in favor of oxidants (46). The MDA levels significantly increased in the lung tissue in both AKI models. SOD and CAT are two necessary anti-oxidant enzymes that respectively convert O_2_^−^ into O_2_ and H_2_O_2_ or catalyze H_2_O_2_ into H_2_O and O_2_ (47). Our results showed that these enzymes’ activity was either compensatory increase or used in response to the oxidative stress condition 24 hr after the kidney injury or kidney removal. Various studies implicate oxidative stress in several lung diseases as well as acute lung injury (48, 49). 

Redox stress directly led to activation of TLRs on the immune or non-immune effector cells. In addition, oxidative stress damaged parenchymal and extracellular matrix cells that released danger-associated molecular patterns (DAMPs), which are endogenous ligands for TLRs. Therefore, redox stress promotes overexpression of TLR directly or indirectly (50). Therefore, our studies’ overexpression of TLR2 and TLR4 and oxidative stress synergistically progresses acute lung injury induced by acute kidney injury. 

***Bone marrow stem cells attenuated the severity of acute lung injury induced by acute kidney injury***

Multifactorial new therapies, including cell therapy for acute and chronic kidney damage, lead to reduced mortality rates and treatment costs (3, 51). Mesenchymal stem cells are a biological therapy suitable for cell therapy and repair of damage due to their multipotentiality, proliferation, and high self-renewal capacity (22, 52). In the current study, BMSCs injection (10^6^ cells, IP) improved renal tubular and vascular injury. The level of creatinine and blood urea reduced following improvement of the kidney’s structure. Abdel Aziz *et al*. induced AKI with bilateral renal ischemia and demonstrated that injection of 10^7^ BMSCs improved the renal pathologic damage as well as renal function by decreasing the increased levels of creatinine and urea in the blood (22). 

Current studies confirmed the anti-inflammatory and anti-oxidative effects of stem cells in acute ischemic renal injury (53, 54). In agreement with these studies, our results showed that bone marrow stem cell therapy could reduce MDA levels and increase CAT activity in the renal tissue after bilateral ischemic-reperfusion. Also, gene expression of TLR2, TLR4, and TNF-α was down-regulated after administration of BMSCs. Therapeutic effects of mesenchymal stem cells have been indicated in acute lung injury induced by endotoxin, bleomycin, and radiation(55). In the current study, administration of BMSCs decreased MDA content and increased CAT activity in lung tissue of the BIR+BMSCs and BNX+BMSCs groups. In addition, mRNA expression of TLR2 and TLR4 decreased in these groups. Liu *et al*. showed that lung expression levels of TLR4 and NF-κB increased after intestinal ischemic reperfusion, and injection of 3×10^6^ BMSCs improved lung injury through TLR4 /NF-κB(53). 

## Conclusion

Kidney and lung interaction is created due to production of injurious molecules or impairing the clearance of systemic mediators. Therefore, gathering of systemic mediators after AKI in the absence of kidneys or kidney injury induces ALI through overexpression of TLR2 and TLR4 as well as oxidative stress. Bone marrow stem cell therapy suppressed acute kidney injury through anti-inflammatory and anti-oxidative pathways. Moreover, the lung protective effect of BMSCs may be related to modulation of TLR2 and TLR4 and oxidative stress in the kidney and lung tissue.

## Authors’ Contributions

ZK, SSH, and SJ Designed the experiments; ZK and SJ Performed experiments and collected data; ZK, SSH, and SJ Discussed the results and strategy; ZK Supervised, directed and managed the study; ZK, SSH, and ZK Final approved of the version to be published (the names of all authors must be listed).

## Conflicts of Interest

The authors declare no conflicts of interest.
